# Has increased clinical experience with methotrexate reduced the direct costs of medical management of ectopic pregnancy compared to surgery?

**DOI:** 10.1186/1471-2393-12-98

**Published:** 2012-09-17

**Authors:** Daniel T Westaby, Olivia Wu, W Colin Duncan, Hilary OD Critchley, Stephen Tong, Andrew W Horne

**Affiliations:** 1 MRC Centre for Reproductive Health, University of Edinburgh, Queen’s Medical Research Institute, 47 Little France Crescent, Edinburgh, EH16 4TJ, UK; 2 Health Economics and Health Technology Assessment Unit, University of Glasgow, Glasgow, UK; 3 Department of Obstetrics and Gynaecology, University of Melbourne, Melbourne, Australia

**Keywords:** Cost analysis, Ectopic pregnancy, Laparoscopy, Methotrexate

## Abstract

**Background:**

There is a debate about the cost-efficiency of methotrexate for the management of ectopic pregnancy (EP), especially for patients presenting with serum human chorionic gonadotrophin levels of >1500 IU/L. We hypothesised that further experience with methotrexate, and increased use of guideline-based protocols, has reduced the direct costs of management with methotrexate.

**Methods:**

We conducted a retrospective cost analysis on women treated for EP in a large UK teaching hospital to (1) investigate whether the cost of medical management is less expensive than surgical management for those patients eligible for both treatments and (2) to compare the cost of medical management for women with hCG concentrations 1500–3000 IU/L against those with similar hCG concentrations that elected for surgery. Three distinct treatment groups were identified: (1) those who had initial medical management with methotrexate, (2) those who were eligible for initial medical management but chose surgery (‘elected’ surgery) and (3) those who initially ‘required’ surgery and did not meet the eligibility criteria for methotrexate. We calculated the costs from the point of view of the National Health Service (NHS) in the UK. We summarised the cost per study group using the mean, standard deviation, median and range and, to account for the skewed nature of the data, we calculated 95% confidence intervals for differential costs using the nonparametric bootstrap method.

**Results:**

Methotrexate was £1179 (CI 819–1550) per patient cheaper than surgery but there were no significant savings with methotrexate in women with hCG >1500 IU/L due to treatment failures.

**Conclusions:**

Our data support an ongoing unmet economic need for better medical treatments for EP with hCG >1500 IU/L.

## Background

Ectopic pregnancy is a common cause of morbidity and occasional mortality in women of reproductive age [1]. It is managed expectantly, medically (with methotrexate) or surgically. Medical management of ectopic pregnancy is generally considered to be less expensive than surgery [2-5].

Expectant management is a conservative strategy that involves careful monitoring and assessment to establish whether the ectopic pregnancy will continue to resolve without the need for intervention. This conservative approach can only be used if the patient is stable, asymptomatic, at low risk of rupture and has decreasing serum human chorionic gonadotrophin (hCG) concentrations [6].

Medical management of ectopic pregnancy may be offered to patients with minimal symptoms, who are haemodynamically stable, have no more than a moderate amount of intra-abdominal free fluid on ultrasound scan and have an hCG concentration <3000 IU/L [6]. Medical management involves the intramuscular administration of methotrexate, usually in a single dose. After receiving methotrexate, patients require close monitoring until their serum hCG drops below 5 IU/l [7]. Around 15-20% of women receiving a single dose of methotrexate will require a repeat dose of this chemotherapeutic agent [7].

Surgical management is indicated if the patient is not eligible for methotrexate or has symptoms or signs of tubal rupture. This usually involves a salpingectomy but a salpingotomy can be used, particularly when there is not a healthy contralateral tube, in an attempt to preserve fertility [6]. In the absence of acute haemodynamic compromise a laparoscopic approach is preferable to an open approach and has been shown to be considerably cheaper [5,8].

Although there have been many studies evaluating the efficacy of the above management options, there are only a small number of studies that have evaluated the cost of treating ectopic pregnancy. Studies from the USA, Canada, New Zealand and France concluded that methotrexate had the potential to lead to significant savings if used as an alternative to laparoscopic surgery [2-4]. However, it has been suggested that management with methotrexate is only cheaper than laparoscopic surgery when used for the treatment of patients with serum hCG concentrations <1500 IU/l [2,5].

We hypothesised that further clinical experience with methotrexate, and the increasing use of guideline-based protocols, has reduced the cost of medical management. Using data from a large UK teaching hospital, we examined (1) whether the cost of medical management of ectopic pregnancy remained overall less expensive than surgical management for those patients that elected for surgery (despite being eligible for methotrexate) and (2) compared the cost of medical management for women with hCG concentrations 1500–3000 IU/L against those with similar hCG concentrations that elected for surgery.

## Methods

### Ethical approval

The study was discussed with the chair of Scotland A Research Ethics Committee and we were advised that no ethical approval was required for this project because it was seen as service evaluation, not research.

### Study population and data collected

We conducted a retrospective analysis on all women treated either medically or surgically for tubal ectopic pregnancy at the RIE between January 2010 and February 2011. Women managed expectantly or where the diagnosis was not clear were excluded. All women had completed their treatment at least one month prior to data collection. Management criteria and eligibility for methotrexate followed national UK guidelines and was strictly assessed as part of an Integrated Care Pathway [5]. All women suitable for methotrexate were offered methotrexate or the possibility of laparoscopic surgery during discussion with their physician. We identified three distinct treatment groups: (1) those who had initial medical management with methotrexate, (2) those who were eligible for initial medical management but chose surgery (‘elected’ surgery) and (3) those who initially ‘required’ surgery and did not meet the eligibility criteria for methotrexate.

For each subject we recorded a comprehensive management pathway including any resources used. Any resource recorded was directly related to the treatment of ectopic pregnancy and no other condition. These data included any outpatient appointments, hospital inpatient admissions, general practice (GP) appointments, surgery, ultrasound scans, methotrexate administration, haematological investigation, biochemical investigation and microbiological investigations. Age, BMI, postcode and serum hCG concentration at diagnosis were also recorded. We calculated the postcode deprivation using the Scottish Index of Multiple Deprivation (SIMD) The SIMD ranks small areas (postcodes) from most deprived – ranked 1 – to least deprived – ranked 6,505, across all of Scotland in a consistent way ( http://www.scotland.gov.uk).

### Data Source

We sourced data using the comprehensive paperless RIE Pregnancy Support Clinic (PSC) records and database. In addition, the hospital-wide database TRAK™ database was interrogated for information about hospital visits outside PSC. We obtained information regarding GP visits from a combination of the PSC database and TRAK. The data were cross-referenced with the operating theatre record books to ensure all emergency surgery was accurately identified.

We calculated the costs from the point of view of the National Health Service (NHS) in the UK. As a result only costs directly attributable to the NHS were included, excluding such costs incurred by patient travel and time off work. We sourced unit costs using Information Service Division (ISD) Scotland prices ( http://www.isdscotland.org) for April 2009 to March 2010, specific for the RIE. The ISD did not have a cost for a serum hCG measurement or methotrexate and therefore we sourced these costs from the RIE laboratory price list 2010–11. Laboratory and theatre costs were calculated specific to each patient. ISD present a breakdown of costs for inpatient admissions (including laboratory and theatre costs). Consequently, we subtracted these two costs from the ISD overall inpatient cost and added our more accurate ‘micro-costing’ to produce an overall net value. Although ISD has a cost for nurse-led clinic outpatient appointments, it does not have a breakdown. As a result, we calculated the cost for a PSC appointment (nurse-led clinic) using the Personal Social Services Research Unit (PSSRU) publication ‘Unit Costs of Health and Social Care 2010’ ( http://www.pssru.ac.uk). This provided an hourly cost for a staff-nurse and allowed us to accurately add the cost of any resource used during the consultation (e.g. ultrasound, laboratory investigation, methotrexate injection). The average time per nurse consultation was calculated at 15 minutes using information from those conducting the appointments. If the patient received an ultrasound scan, the appointment time was estimated at 30 minutes.

All prices used in this study include hospital overhead costs (e.g. administration, housekeeping, laundry). We calculated the direct costs for each patient by multiplying the number of each resource used by the unit cost of the resource. The total cost for each resource was then added together to give an overall cost for the patient. All costs were calculated at 2009 values (UK £).

### Data Analysis

Cost data are usually right-skewed. Faced with skewed data one would normally use the median as a measure of the distribution, along with the range. However, with a cost analysis it is important to link the summary measure of cost per patient to the overall budget impact. The only way to achieve this is to use the mean. Therefore, we summarised the cost per study group using the mean, standard deviation, median and range, and to account for the skewed nature of the data, we calculated 95% confidence intervals for differential costs using the nonparametric bootstrap method. The 95% percentile confidence intervals are based on 1000 bootstrap replications.

We collected postcode and age for all patients with no missing data. Age showed a normal distribution across the three groups (Shapiro-Wilk: *P* >0.05) and as a result an independent samples student *t*-test was performed. Postcode score was not normally distributed (Shapiro-Wilk: *P* <0.05) and we used a Chi-Squared analysis to test whether there was a relationship between postcode score and management choice for those who were eligible for both treatments. The frequencies were weighted before the test was performed. We compared the proportion of failed methotrexate treatment where the initial hCG concentrations were <1500 IU/l or ≥1500 IU/l using Fisher’s exact test.

All statistical analysis was carried out using IBM® SPSS® 17. The alpha level was set at α = 0.05 for all statistical tests, equating to a 95% confidence level.

## Results

### Demographics

During the study period (14 months), 129 patients were treated for ectopic pregnancy at the Royal Infirmary of Edinburgh (RIE), Edinburgh, UK. Two patients with non-tubal ectopic pregnancies were excluded after data collection (a caesarean section scar ectopic and cervical ectopic) as the management protocols differ in these atypical cases. Thus, we analysed data from 127 patients: 59 were ineligible for medical management and were managed surgically, 20 were eligible for medical management but opted for initial surgical management and 48 were initially managed medically using systemic methotrexate. All surgery was performed laparoscopically. There was no significant difference in age between the medically managed group and elective surgery group (t = −0.029, df = 66, *P > 0.05)*, and between the elective surgery and the ‘requiring surgery’ groups (t = 0.859, df = 77, *P > 0.05)*. Statistical analysis on body mass index (BMI) was not possible due to missing data (60/129 missing). However, a descriptive comparison suggested the findings were similar across all three groups although no definitive conclusion may be drawn (Table 1). In addition, there were no differences in the postcode deprivation scores of the women eligible for methotrexate that opted for initial surgery when compared to those treated initially with methotrexate.

**Table 1:** Baseline Characteristics categorised by patient management group

	**Methotrexate**		**Elective surgery**		**Required surgery**	
Age (mean ± SD)	31.5 ± 5.6		31.5 ± 4.8		30.2 ± 6.0	
BMI (median/range)	24.5	17.9-41.4	23.9	19.7-29.9	25.2	18.7-43.8

### Outcomes and use of resources

The patients who received methotrexate were monitored carefully to ensure complete resolution of the ectopic gestation using serial assessment of hCG levels every 4–7 days until hCG <5 IU/L. We defined treatment failure as those women in whom the physician responsible for the care opted for surgical management after methotrexate treatment. This was either due to failure of the hCG to fall, symptoms such as significant pain or clinical features of possible tubal rupture or intraperitoneal haemorrhage.

Out of the 48 patients initially medically managed with methotrexate, 13 required surgery (27%) due to treatment failure. Eight women required additional methotrexate (16.7%). Within this group of eight women, seven received one additional dose of methotrexate, one of whom then required subsequent surgery, and one received two additional doses of methotrexate. Seven of the 48 patients medically managed had an initial serum hCG level ≥1500 IU/l. Methotrexate therapy failed in four of these cases (57%) resulting in inpatient admission and surgery. The patients with initial hCG concentrations <1500 IU/l were less likely to require surgery (Fisher’s exact: *P* < 0.05) for failed methotrexate treatment (17%). The 48 women had a total length of stay of 33 days, of which 28.6 days were due to surgery following treatment failure.

The elective surgery group had a longer length of stay in hospital on average (1.35 days) compared to the methotrexate group (0.68 days) (t = 2.447, df = 66, *P* < 0.05). The group requiring surgery, without an option for medical management, had an even longer average inpatient stay (1.91 days) (t = 2.497, df = 77, *P* < 0.05). Two of the 59 women requiring surgical intervention also required an additional methotrexate injection. These women had evidence of persistent trophoblast on serial hCG testing instigated because of trophoblast spillage after tubal rupture, or after salpingotomy.

Women initially treated with methotrexate had on average a far greater number of outpatient PSC appointments (5.77) compared to those opting for ‘elective surgery’ (0.35) (t = 10.858, df = 105, *P* < 0.0001) and those initially ‘requiring surgery’ (0.56) (t = 7.0158, df = 66, *P* < 0.0001). Patients receiving methotrexate are required to return to the PSC regularly until hCG concentrations are <5 IU/l. Patients are monitored at these appointments and this was reflected in the increased haematology, clinical biochemistry and in particular serum hCG measurements (t = 6.549, df = 66, *P* < 0.0001) and additional ultrasound scans (t = 2.9632, df = 66, *P* < 0.005) associated with the methotrexate group (Table 2). There was no difference in the number of microbiology specimens requested in the three management groups.

**Table 2:** Resource use and cost categorised by patient management group

**Resource**		**Methotrexate**			**Elected Surgery**			**Required Surgery**		
**(U = Use)**		**(n = 48)**			**(n = 20)**			**(n = 59)**		
**(C = Cost(£))**		**Total**	**Mean**	**SD**	**Total**	**Mean**	**SD**	**Total**	**Mean**	**SD**
PSC Appointments	U	245/32	5.77	3.40	06/01	0.35	0.82	28/05	0.56	1.29
(15 min / 30 min)	C	1777.75	37.02	21.61	46.00	2.30	4.72	218.50	3.70	8.59
Inpatient care	U	33.00	0.68	1.16	26.93	1.35	0.59	112.60	1.91	0.94
(Days)	C	17030.36	354.79	603.29	14003.89	700.19	305.83	58554.52	992.45	490.03
Surgery	U	13.00	0.27	0.45	20.00	1.00	0.00	59.00	1.00	0.00
(Operations)	C	17524.78	365.10	605.40	26961.20	1348.06	0.00	79535.54	1348.06	0.00
Methotrexate	U	57.00	1.19	0.45	0.00	0.00	0.00	2.00	0.03	0.18
(Injections)	C	225.15	4.69	1.76	0.00	0.00	0.00	7.90	0.13	0.72
Serum hCG	U	255.00	5.31	3.50	3.00	0.15	0.37	35.00	0.59	1.59
(Tests)	C	2703.00	56.31	37.12	31.80	1.59	3.89	371.00	6.29	16.83
Ultrasound	U	57.00	1.19	1.53	3.00	0.15	0.50	15.00	0.25	0.63
(Scans)	C	2816.80	58.66	75.34	148.20	7.41	24.17	741.00	12.56	31.23
Haematology	U	242.00	5.04	2.01	46.00	2.25	1.08	167.00	2.83	1.53
(Tests)	C	719.74	14.97	5.97	136.62	6.83	3.21	495.99	8.41	4.55
Biochemistry	U	339.00	7.06	3.94	34.00	1.70	1.92	105.00	1.78	1.51
(Tests)	C	207.79	4.31	2.40	20.74	1.04	1.17	64.05	1.09	0.92
Microbiology	U	75.00	1.56	1.50	33.00	1.65	2.01	104.00	1.76	1.69
(Tests)	C	853.75	17.77	17.06	375.21	18.76	22.82	1182.48	20.04	19.16
A&E	U	1.00	0.02	0.14	1.00	0.05	0.22	0.00	0.00	0.00
(Attendances)	C	124.00	2.58	17.90	124.00	6.20	27.73	0.00	0.00	0.00
GP	U	1.00	0.02	0.14	1.00	0.05	0.22	7.00	0.12	0.56
(Appointments)	C	53.00	1.10	7.65	53.00	2.65	11.85	371.00	6.29	29.67
**Total Cost (£)**		43991.47			41909.81			141568.83		
**Cost per patient (£)**										
**mean ± SD**		916.49 ±1157.70			2095.49 ±353.01			2399.47 ±505.10		
**median (range)**		270.00 (68.88-3948.58)			1922.30 (1738.80-3011.59)			2403.38 (1822.59-4460.59)		

### Cost analysis of treatment

The resource costs for each patient management group are also shown in Table 2. The mean cost per patient in the initially medically managed group was £916.49 ± 1157.70 (median = £270.00, range = £68.88 – £3948.58). In the ‘elected’ surgery group, the mean cost per patient was £2095.49 ± 353.01 (median = £1922.30, range = £1738.80 – £3011.59). Finally, in the ‘required’ surgery group the mean cost per patient was £2399.47 ± 505.10 (median = £2403.38, range = £1822.59 - £4460.59). Of those suitable for either methotrexate or surgery, methotrexate treatment was £1179 (CI 819–1550) cheaper than surgery (reflecting potential savings per patient).

Surgery, followed by inpatient admissions, had the most substantial impact on cost, accounting for 40% and 39% respectively of the methotrexate group cost, 64% and 33% respectively for the ‘elected’ surgery group cost and 56% and 41% respectively of the ‘required’ surgery group cost. The group that initially required surgery (ie not eligible for methotrexate) cost significantly more than the group who were suitable for methotrexate but elected for surgery (Mann Whitney: U = 347, *P* < 0.05). The apparent reason for this increased expense was an increased inpatient stay post operation (Table 2). These patients were also more likely to have PSC follow up appointments for post procedural serial hCG measurements (Fisher’s exact: *P* < 0.05).

For those medically managed with an initial serum hCG <1500 IU/l (n = 41) the mean cost per patient (£813.06 ± 1152.30, median = £169.80, range = £68.88 - £3948.58) was significantly lower (Mann Whitney: U = 71, *P* < 0.05) than those medically managed with an serum hCG between 1500 and 3000 IU/L (n = 7) (£1522.27 ± 1070.33, median = £1991.10, range = £315.45 - £2923.11). This difference was accounted for by an increased methotrexate failure rate and the need for subsequent surgical intervention along with inpatient admission. Where patients had an initial serum hCG between 1500 and 3000 IU/l, the difference in costs between those managed in the elective surgery group (£2045.16 ± 363.07, median = £1869.83, range = £1738.80 - £2679.78) and those initially managed medically (£1522.27 ± 1070.33, median = £1991.10, range = £315.45-£2923.11) was not significant (Mann Whitney: U = 22, *P* > 0.05).

The cost for successful methotrexate treatment was significantly less (n = 35; mean = £255.67 ±251.75; median = £159.01, range = £68.88 - £1334.26) than the cost for failed methotrexate treatment where surgery is required (n = 13; mean = £2695.61 ± 605.91, median = £2503.79, range = £1983.50 - £3948.58) (Mann Whitney: U = 0, *P* < 0.05). It is clear when comparing elective and required surgery costs that this difference could be explained by the added cost of surgery and subsequent inpatient admission time. All three groups showed right-skewed data reflecting a small proportion of patients with notably high costs. This group of patients reflect complications with treatment or initial treatment failure and explain the large standard deviations reported, particularly within the medically managed group.

## Discussion

To our knowledge, this is the first cost-analysis comparing medical and surgical management of ectopic pregnancy performed in the UK Healthcare system. The results support the general consensus that medical management of ectopic pregnancy remains economically more viable than surgical management for those patients eligible for both treatment approaches.

Earlier analysis in other health-care settings suggested that methotrexate is more expensive than laparoscopic surgery when the initial hCG is >1500 IU/L [2,5]. Mol *et al*’s review from 2008 concludes that laparoscopic surgery is the most cost–effective treatment for tubal EP. However, both economic studies cited [2,3] show cost savings for medical management in patients with an initial hCG <1500 IU/L. In the large teaching hospital used for our study only 7 out of 48 patients managed medically presented with an initial serum hCG > 1500 IU/L. Although guidelines do not rule out medical management for these patients it is clear other factors and clinician judgement often lead to surgical management.

Although we hypothesised that increased experience with methotrexate and the increasing use of protocols developed from evidence-based guidelines [5] would have reduced the costs of medical management, our findings also suggest that medical management may not be less expensive than surgery for patients with initial serum hCG concentrations between 1500 and 3000 IU/l. This is due to the occurrence of treatment failure and is in agreement with Mol *et al’*s 1999 metanalysis of older economic evaluations of managements for ectopic pregnancy [2]. Although the different healthcare systems may have different costs it is likely that cost differentials are similar and that our findings are applicable to other health care settings.

One of the main strengths of our study is that we performed a comprehensive detailed retrospective analysis of all patients managed in a specified time period. There were no missing data values and all direct UK health care costs were taken into account. The cost of post-treatment complications and secondary treatment were included, making this study comprehensive and realistic in reflecting the cost to UK health services. We had similar age, BMI and deprivation index baseline characteristics for each treatment group. A further strength was that, where possible, we used national hospital-cost databases (ISD Scotland, PSSRU) allowing hospitals across the UK to relate to the findings. Any costs not sourced from these databases were calculated with advice from a health economist.

On the other hand, the study has some limitations. We only took into account direct UK healthcare costs. Indirect costs, such as travel expenses and time off work, are undoubtedly important. In addition, we did not analyse the costs of conservative management although we predict that the costs may not be less than successful methotrexate treatment. Furthermore, we decided to focus solely on costs in this study but it is important to acknowledge that health benefits and quality of life are also extremely relevant. For example, if women in the surgery group have a better quality of life than the methotrexate group, this may be a more cost effective option overall.

## Conclusions

The clear message from this study is that it is the cost of treatment failure and subsequent surgery that makes methotrexate less cost-effective in women with hCG concentrations of >1500 IU/L. This finding is still the same after over 10 years of increasing experience and more standardised treatment. This means that, rather than minor monitoring or follow-up changes, there are two important areas for research to improve the cost-effectiveness of medical management of ectopic pregnancy. The first is the identification of clinical features or biomarkers predictive of methotrexate success and the second is the use of additional medical treatments or novel adjuncts that reduce treatment failures.

## Competing interests

AWH and HODC hold a UK patent for a diagnostic biomarker for ectopic pregnancy (# 0712801.0). AWH is Medical Advisor to the Ectopic Pregnancy Trust.

## Authors’ contributions

DTW and OW were responsible for data analysis and manuscript preparation. WCD, HODC and ST were responsible for manuscript preparation. AWH conceived the project, and was responsible for data analysis and manuscript preparation. All authors read and approved the final manuscript.

## Pre-publication history

The pre-publication history for this paper can be accessed here:

http://www.biomedcentral.com/1471-2393/12/98/prepub
